# Supplementary material for: Baseline Circulating Blood Cell Counts and Ratios and Changes Therein for Predicting Immune-Related Adverse Events during Immune Checkpoint Inhibitor Therapy: A Multicenter, Prospective, Observational, Pan-Cancer Cohort Study with a Gender Perspective
Source: Cancers (Basel). 2023 Dec 28;16(1):151. doi: 10.3390/cancers16010151 (PMC10778233; doi:10.3390/cancers16010151)
Supplement: Supplementary file 1 [file cancers-16-00151-s001.zip › cancers-2778889-supplementary.pdf]

Supplementary Table S1. Summary of the immune checkpoint inhibitors used and their tumor indications at the time of the design of the study.

Treatment	Target	Tumor indication
Nivolumab monotherapy	PD-1	Adjuvant therapy for esophageal cancer<br>Adjuvant therapy for melanoma<br>First-line treatment for melanoma<br>Platinum-refractory urothelial carcinoma<br>Second-line treatment for renal carcinoma<br>Platinum-refractory head and neck cancer<br>Second-line treatment for non-small-cell lung carcinoma
Pembrolizumab monotherapy	PD-1	Adjuvant therapy for melanoma<br>First-line treatment for melanoma<br>First- or subsequent-line treatment for non-small cell lung carcinoma<br>First- or subsequent-line treatment for urothelial carcinoma<br>First-line treatment for head and neck cancer<br>Second-line treatment for mismatch repair deficient tumors
Cemiplimab monotherapy	PD-1	First-line treatment for non-small-cell lung cancer<br>Second-line treatment for cervical cancer
Avelumab monotherapy	PD-L1	First-line treatment for Merkel cell carcinoma<br>First-line treatment for urothelial carcinoma
Atezolizumab monotherapy	PD-L1	Adjuvant therapy for non-small-cell lung cancer,<br>First or subsequent lines treatment for non-small-cell lung carcinoma<br>First-line and subsequent-line treatment for urothelial carcinoma
Durvalumab monotherapy	PD-L1	Locally advanced non-small-cell lung carcinoma
Ipilimumab plus nivolumab	CTLA-4 + PD-1	First-line treatment for melanoma<br>First-line treatment for mesothelioma<br>First-line treatment for urothelial carcinoma<br>Second-line treatment for mismatch repair deficient colorectal carcinoma

Abbreviations in alphabetical order: CTLA-4, cytotoxic T-lymphocyte-associated antigen-4; PD-1, programmed cell death protein-1; PD-L1, programmed cell death protein-1 ligand 1.

Supplementary Table S2. Cumulative incidence of immune-related adverse events over time using a Kaplan–Meier method and a Fine and Gray competing risk method.

	Kaplan–Meier method	Fine and Gray Competing Risk method	
	irAE (95% CI)	irAE (95% CI)	Death (95% CI)
At 30 days	12.3% (6.60-17.5)	11.9% (7.2-17.8)	4.9% (2.1-9.3)
At 90 days	28.5% (20.1-36.1)	25.9% (18.9-33.4)	21.3% (14.9-28.5)
At 1 year	52.0% (39.6-61.9)	41.6% (32.6-50.4)	32.9% (24.3-41.7)

Abbreviations in alphabetical order: CI, confidence interval; irAE, immune-related adverse event.
Analyses performed using a Kaplan–Meier method and a Fine and Gray competing risk method with death as the competing event.

Supplementary Table S3. Summary of all immune-related adverse events in the cohort patients*.

ID	Type of tumor	ICI type	Total number of irAEs	First irAE			Second irAE			Third irAE		
				Type	Grade†	Time‡	Type	Grade†	Time‡	Type	Grade†	Time‡
2	HNSCC	Nivolumab	1	Hemolytic anemia	2	42	-	-	-	-	-	-
3	HNSCC	Nivolumab	1	Thyroiditis	2	14	-	-	-	-	-	-
4	HNSCC	Nivolumab	3	Nephritis	2	79	Nephritis	2	270	Arthromyalgia	2	270
5	NSCLC	Durvalumab	3	Maculopapular rash	1	72	Pneumonitis	2	268	Arthromyalgia	1	410
6	NSCLC	Nivolumab	1	Pneumonitis	2	111	-	-	-	-	-	-
8	CRA	Pembrolizumab	2	Lupus flare	1	25	Maculopapular rash	1	266	-	-	-
9	GA	Ipi–Nivo	1	Mucositis	1	155	-	-	-	-	-	-
10	RCC	Ipi–Nivo	5	Maculopapular rash	2	186	Maculopapular rash	1	428	Polymyalgia rheumatica	1	449
11	Melanoma	Pembrolizumab	1	Colitis	1	43	-	-	-	-	-	-
14	HNSCC	Nivolumab	3	Hyperthyroidism	1	28	Nephritis	2	105	Pruritus	2	105
27	HNSCC	Nivolumab	1	Maculopapular rash	1	91	-	-	-	-	-	-
28	NSCLC	Nivolumab	1	Hypothyroidism	2	42	-	-	-	-	-	-
29	MPM	Ipi–Nivo	2	Colitis	2	56	Pneumonitis	2	122	-	-	-
30	RCC	Ipi–Nivo	1	Adrenal insufficiency	2	31	-	-	-	-	-	-
34	GA	Pembrolizumab	2	Colitis	2	46	Hepatitis	1	109	-	-	-
35	RCC	Ipi–Nivo	1	Maculopapular rash	1	21	-	-	-	-	-	-
40	UC	Avelumab	2	Psoriasiform rash	2	29	Arthritis	3	29	-	-	-
41	RCC	Ipi–Nivo	1	Eczema	1	23	-	-	-	-	-	-
45	RCC	Ipi–Nivo	1	Hepatitis	3	43	-	-	-	-	-	-
48	HNSCC	Pembrolizumab	1	Maculopapular rash	2	46	-	-	-	-	-	-
49	UC	Atezolizumab	1	Maculopapular rash	1	112	-	-	-	-	-	-
55	UC	Atezolizumab	1	Hyperthyroidism	1	52	-	-	-	-	-	-
57	UC	Atezolizumab	1	Hyperthyroidism	2	97	-	-	-	-	-	-
58	HNSCC	Nivolumab	1	Maculopapular rash	1	14	-	-	-	-	-	-
59	NSCLC	Pembrolizumab	1	Maculopapular rash	1	21	-	-	-	-	-	-
60	Melanoma	Nivolumab	1	Maculopapular rash	1	49	-	-	-	-	-	-
63	HNSCC	Nivolumab	1	Hypertrichosis	1	28	-	-	-	-	-	-
64	HNSCC	Nivolumab	2	Pancytopenia	1	14	Hypothyroidism	1	56	-	-	-
65	RCC	Ipi–Nivo	1	Inflammatory arthritis	1	10	-	-	-	-	-	-
68	NSCLC	Durvalumab	1	Maculopapular rash	1	27	-	-	-	-	-	-
70	UC	Atezolizumab	1	Colitis	1	20	-	-	-	-	-	-
73	NSCLC	Pembrolizumab	1	Nephritis	2	169	-	-	-	-	-	-
81	NSCLC	Atezolizumab	1	Arthromyalgia	2	21	-	-	-	-	-	-

87	NSCLC	Pembrolizumab	2	Inflammatory arthritis	1	150	Nephritis	2	184	-	-	-
89	UC	Atezolizumab	1	Hypothyroidism	2	150	-	-	-	-	-	-
90	NSCLC	Atezolizumab	1	Hypothyroidism	2	179	-	-	-	-	-	-
91	NSCLC	Pembrolizumab	3	Hyperthyroidism	1	62	Hypothyroidism	1	251	Adrenal insufficiency	2	259
94	UC	Avelumab	1	Maculopapular rash	1	128	-	-	-	-	-	-
98	RCC	Nivolumab	1	Uveitis	2	113	-	-	-	-	-	-
102	NSCLC	Pembrolizumab	1	Pruritus	1	46	-	-	-	-	-	-
107	Melanoma	Ipi–Nivo	5	Hepatitis	3	84	Vitiligo	1	182	Pruritus	1	196
108	Melanoma	Pembrolizumab	1	Polymyalgia rheumatica	2	43	-	-	-	-	-	-
109	HNSCC	Nivolumab	1	Hypothyroidism	2	176	-	-	-	-	-	-
112	RCC	Ipi–Nivo	2	Thyroiditis	1	60	Encephalitis	1	109	-	-	-
113	RCC	Ipi–Nivo	1	Thyroiditis	1	60	-	-	-	-	-	-
121	Melanoma	Pembrolizumab	1	Neutropenia	2	60	-	-	-	-	-	-
123	RCC	Ipi–Nivo	4	Maculopapular rash	1	15	Hepatitis	2	36	Hyperthyroidism	1	45
124	Melanoma	Ipi–Nivo	2	Hyperthyroidism	2	10	Pruritus	1	41	-	-	-
129	RCC	Nivolumab	1	Nephritis	3	72	-	-	-	-	-	-
139	RCC	Ipi–Nivo	1	Hypothyroidism	1	23	-	-	-	-	-	-
141	RCC	Pembrolizumab	1	Pruritus	1	42	-	-	-	-	-	-
142	RCC	Ipi–Nivo	1	Arthromyalgia	2	63	-	-	-	-	-	-

Abbreviations in alphabetical order: CRA, colorectal adenocarcinoma; GA, gastric adenocarcinoma; HNSCC, head and neck squamous cell carcinoma; ICI, immune checkpoint inhibitor; ID, patient identification number; Ipi–Nivo, ipilimumab plus nivolumab; irAE, immune-related adverse event; MPM, malignant pleural mesothelioma; NSCLC, non-small-cell lung cancer; RCC, renal cell carcinoma; UC, urothelial carcinoma.

* For representative purposes, only the first three events are presented.

† According to Common Terminology Criteria for Adverse Events v. 5.0.

‡ Time from ICI initiation to irAE diagnosis expressed in days.

Supplementary Table S4. Blood cell parameters under study at baseline (pre-first ICI cycle) and after the first ICI cycle (post-first ICI cycle) and their relative increase between pre- and post-first ICI cycle blood sampling in the 134 patients who reached the second ICI cycle without being censored.

Parameter	Pre-first ICI cycle*	Post-first ICI cycle*	Relative increase*†	p-value‡
WBC, K/μL	7.57 ± 3.08	7.53 ± 2.68	0.05 ± 0.33	0.455
ANC, K/μL	5.13 ± 2.86	4.84 ± 2.38	0.05 ± 0.47	0.034
ALC, K/μL	1.54 ± 0.72	1.68 ± 0.80	0.18 ± 0.55	0.017
AMC, K/μL	0.67 ± 0.26	0.72 ± 0.26	0.13 ± 0.40	0.020
AEC, K/μL	0.19 ± 0.17	0.23 ± 0.20	0.04 ± 0.18	0.008
PC, K/μL	269.40 ± 97.14	283.07 ± 106.06	0.07 ± 0.26	0.034
NLR	4.53 ± 5.27	3.62 ± 2.73	0.04 ± 0.70	0.03
dNLR	0.83 ± 0.06	0.81 ± 0.07	-0.02 ± 0.08	< 0.001
MLR	0.54 ± 0.35	0.51 ± 0.29	0.05 ± 0.40	0.143
ELR	0.14 ± 0.14	0.16 ± 0.17	0.02 ± 0.14	0.106
PLR	224.37 ± 172.13	206.79 ± 121.22	0.04 ± 0.46	0.097

Abbreviations in alphabetical order: AEC; absolute eosinophil count; ALC; absolute lymphocyte count; AMC, absolute monocyte count; ANC, absolute neutrophil count; dNLR, derived neutrophil-to-lymphocyte ratio (calculated as ANC/(WBC – ALC)); ELR, eosinophil-to-lymphocyte ratio (calculated as AEC/ALC); ICI, immune checkpoint inhibitor; K/μL, thousand cells per microliter; MLR, monocyte-to-lymphocyte ratio (calculated as AMC/ALC); NLR, neutrophil-to-lymphocyte ratio (calculated as ANC/ALC); PC, platelet count; PLR, platelet-to-lymphocyte ratio (calculated as PC/ALC); WBC, white blood cell count.

* Data expressed as mean ± standard deviation.

† Calculated as (post-first ICI cycle − pre-first ICI cycle)/pre-first ICI cycle.

‡ Estimated between pre- and post-first ICI cycle using a Wilcoxon signed-rank test for paired data.

Supplementary Table S5. Values of blood cell parameters under study at baseline (pre-first ICI cycle) and after the first ICI cycle (post-first ICI cycle) by patient characteristics.

		**Age at inclusion**		**Sex**		**Pre-existing autoimmune disease**		**ICI therapy modality**	
		**< 65 years (n = 60)**	**≥ 65 years (n = 74)**	**Men (n = 105)**	**Women (n = 29)**	**No (n = 125)**	**Yes (n = 9)**	**Monotherapy (n =113)**	**Dual therapy (n = 21)**
WBC, K/μL	Pre-first ICI cycle	7.17 ± 2.78	7.89 ± 3.28	7.62 ± 3.13	7.39 ± 2.95	7.70 ± 3.13	5.77 ± 1.31	7.60 ± 3.23	7.40 ± 2.12
	Post-first ICI cycle	7.38 ± 2.89	7.65 ± 2.51	7.66 ± 2.50	7.05 ± 3.26	7.64 ± 2.67	6.02 ± 2.48	7.50 ± 2.71	7.68 ± 2.53
ANC, K/μL	Pre-first ICI cycle	4.70 ± 2.67	5.48 ± 2.97	5.19 ± 2.90	4.88 ± 2.72	5.24 ± 2.92	3.54 ± 0.89	5.20 ± 3.00	4.73 ±1.88
	Post-first ICI cycle	4.76 ± 2.76	4.90 ± 2.03	4.92 ± 2.16	4.55 ± 3.07	4.93 ± 2.40	3.65 ± 1.72	4.91 ± 2.41	4.48 ± 2.20
ALC, K/μL	Pre-first ICI cycle	1.48 ± 0.67	1.48 ± 0.67	1.49 ± 0.67	1.70 ± 0.88	1.54 ± 0.72	1.45 ± 0.78	1.49 ± 0.73	1.80 ± 0.64
	Post-first ICI cycle	1.63 ± 0.67	1.71 ± 0.90	1.68 ±0.84	1.67 ± 0.65	1.68 ± 0.81	1.56 ± 0.77	1.60 ± 0.77	2.06 ± 0.85
AMC, K/μL	Pre-first ICI cycle	0.62 ± 0.22	0.71 ± 0.28	0.69 ± 0.27	0.61 ± 0.23	0.68 ± 0.26	0.60 ± 0.23	0.67 ± 0.26	0.67 ± 0.22
	Post-first ICI cycle	0.68 ± 0.24	0.75 ± 0.26	0.56 ± 0.35	0.62 ± 0.26	0.72 ± 0.25	0.62 ± 0.30	0.72 ± 0.26	0.70 ± 0.24
AEC, K/μL	Pre-first ICI cycle	0.19 ± 0.16	0.19 ± 0.18	0.20 ± 0.17	0.16 ± 0.17	0.19 ± 0.17	0.13 ± 0.12	0.19 ± 0.18	0.16 ± 0.08
	Post-first ICI cycle	0.22 ± 0.20	0.23 ± 0.21	0.26 ± 0.22	0.12 ± 0.07	0.23 ± 0.21	0.17 ± 0.13	0.21 ± 0.18	0.34 ± 0.27
PC, K/μL	Pre-first ICI cycle	268.23 ± 76.20	270.34 ± 111.78	259.90 ± 77.59	303.76 ± 144.55	269.62 ± 99.73	266.33 ± 51.77	261.96 ± 79.90	309.38 ± 158.11
	Post-first ICI cycle	280.27 ± 108.64	280.27 ± 108.64	266.14 ± 87.74	344.38 ± 140.95	285.92 ± 107.13	243.56 ± 84.99	277.81 ± 105.75	311.38 ± 105.74
NLR	Pre-first ICI cycle	3.91 ± 4.29	5.03 ± 5.93	4.72 ± 5.66	3.84 ± 3.53	4.62 ± 5.42	3.20 ± 1.92	4.81 ± 5.64	3.01 ± 1.89
	Post-first ICI cycle	3.44 ± 2.40	3.77 ± 2.97	3.77 ± 2.78	3.10 ± 2.49	3.69 ± 2.79	2.65 ± 1.47	3.85 ± 2.85	2.41 ± 1.47
dNLR	Pre-first ICI cycle	0.82 ± 0.07	0.84 ± 0.54	0.83 ± 0.06	0.84 ± 0.06	0.83 ± 0.06	0.83 ± 0.04	0.83 ± 0.06	0.83 ± 0.06
	Post-first ICI cycle	0.80 ± 0.08	0.82 ± 0.06	0.81 ± 0.07	0.81 ± 0.08	0.81 ± 0.07	0.81 ± 0.09	0.82 ± 0.07	0.78 ± 0.08
MLR	Pre-first ICI cycle	0.46 ± 0.26	0.60 ± 0.40	0.56 ± 0.35	0.47 ± 0.33	0.54 ± 0.39	0.54 ± 0.35	0.56 ± 0.36	0.43 ± 0.28
	Post-first ICI cycle	0.60 ± 0.40	0.55 ± 0.33	0.55 ± 0.31	0.40 ± 0.17	0.52 ± 0.29	0.45 ± 0.26	0.54 ± 0.30	0.36 ± 0.12
ELR	Pre-first ICI cycle	0.14 ± 0.15	0.14 ± 0.14	0.15 ± 0.15	0.11 ± 0.13	0.14 ± 0.14	0.12 ± 0.18	0.15 ± 0.15	0.09 ± 0.05
	Post-first ICI cycle	0.16 ± 0.18	0.16 ± 0.17	0.18 ± 0.19	0.08 ± 0.06	0.16 ± 0.17	0.15 ± 0.17	0.16 ± 0.18	0.16 ± 0.11
PLR	Pre-first ICI cycle	207.08 ± 127.51	238.40 ± 200.99	220.21 ± 171.15	239.44 ± 177.86	223.06 ± 175.11	242.68 ± 130.07	227. 59 ± 174.34	207.09 ± 162.64
	Post-first ICI cycle	202.73 ± 103.29	210.08 ± 134.62	199.91 ± 123.17	231.68 ± 112.40	207.19 ± 121.72	201.14 ± 120.85	213.61 ± 125.93	170.06 ± 85.03

Abbreviations in alphabetical order: AEC; absolute eosinophil count; ALC; absolute lymphocyte count; AMC, absolute monocyte count; ANC, absolute neutrophil count; dNLR, derived neutrophil-to-lymphocyte ratio (calculated as ANC/(WBC – ALC)); ELR, eosinophil-to-lymphocyte ratio (calculated as AEC/ALC); ICI, immune checkpoint inhibitor; K/μL, thousand cells per microliter; MLR, monocyte-to-lymphocyte ratio (calculated as AMC/ALC); NLR, neutrophil-to-lymphocyte ratio (calculated as ANC/ALC); PC, platelet count; PLR, platelet-to-lymphocyte ratio (calculated as PC/ALC); WBC, white blood cell count.

All data are expressed as mean ± standard deviation.

Supplementary Table S6. Univariate analysis of potentially explanatory variables available in the study.

Univariate analysis			
**Variable**	**HR**	**95% CI**	**p-value**
Female sex	2.04	1.10-3.70	0.025
Age	0.99	0.97-1.02	0.47
Smoking history	0.51	0.28-0.92	0.026
Lung cancer	0.43	0.23-0.82	0.011
Anti-CTLA-4 (vs. anti-PD-1 or anti-PD-L1 monotherapy)	2.26	1.24-4.10	0.007
Body mass index	0.96	0.91-1.02	0.21
Renal failure	0.30	0.04-2.10	0.23
Pre-first ICI cycle ALC	1.60	1.11-2.31	0.011
Post-first ICI cycle ANC	0.81	0.68-0.95	0.012

Abbreviations in alphabetical order: ALC, absolute lymphocyte count; ANC, absolute neutrophil count; CI, confidence interval; HR, hazard ratio; ICI, immune checkpoint inhibitor.

Analyses performed using a Fine and Gray competing risk model with death as the competing event.

Supplementary Figure S1. Levels of blood cell parameters under study at baseline and after the first ICI cycle.

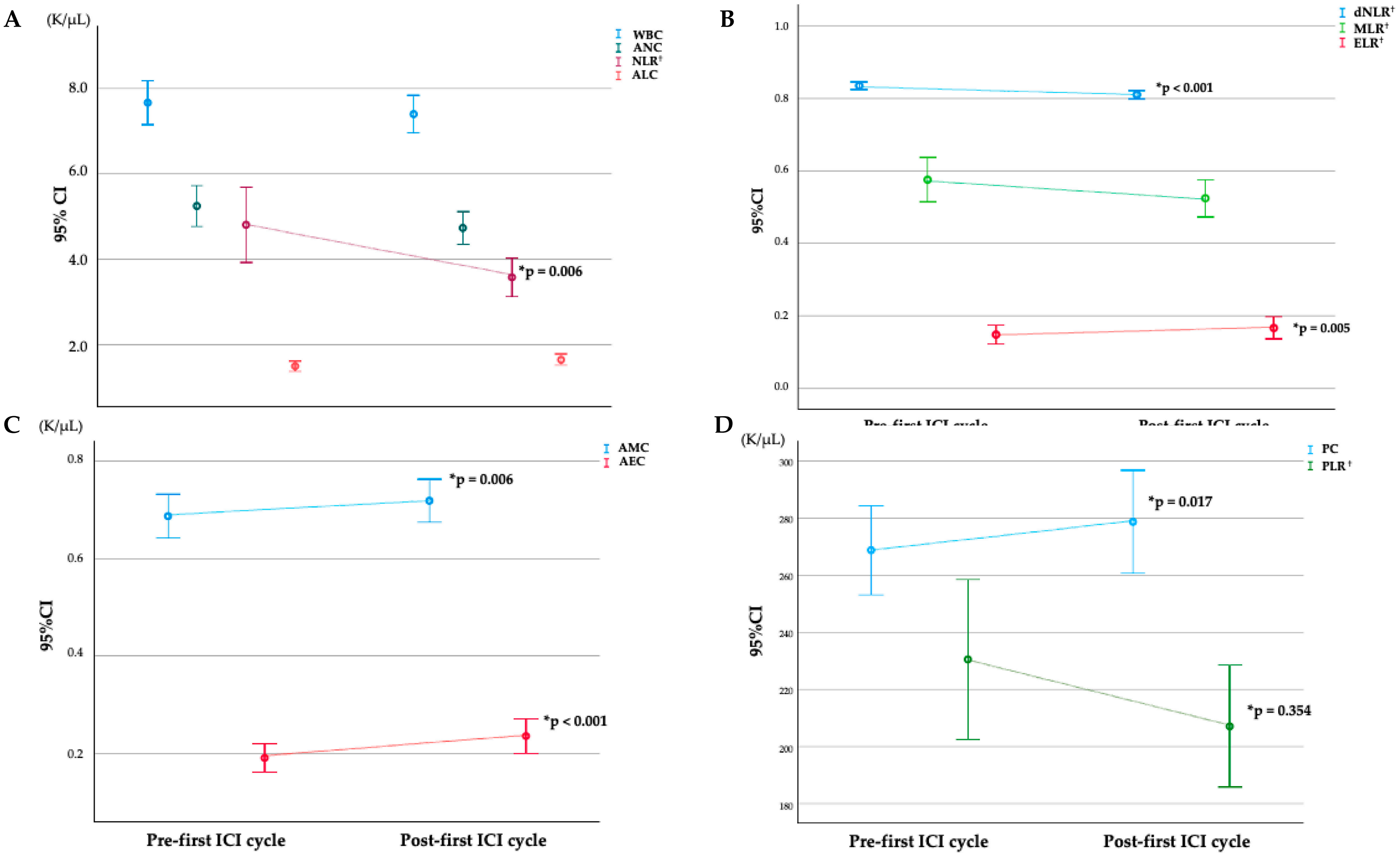

Abbreviations in alphabetical order: AEC, absolute eosinophil count; ALC, absolute lymphocyte count; AMC, absolute monocyte count; ANC, absolute neutrophil count; CI, confidence interval; dNLR, derived neutrophil-to-lymphocyte ratio (calculated as ANC/(WBC − ALC)); ELR, eosinophil-to-lymphocyte ratio (calculated as AEC/ALC); ICI, immune checkpoint inhibitor; MLR, monocyte-to-lymphocyte ratio (calculated as AMC/ALC); NLR, neutrophil-to-lymphocyte ratio (calculated as ANC/ALC); PLR, platelet-to-lymphocyte ratio (calculated as PC/ALC); WBC, white blood cell count.

* Estimated between pre- and post-first ICI cycle using a Wilcoxon signed-rank test for paired data.

† NLR, dNLR, MLR, ELR, and PLR do not have units.

Supplementary Figure S2. White blood cell, absolute neutrophil, and absolute lymphocyte counts at baseline and after the first ICI cycle as a function of patient characteristics.

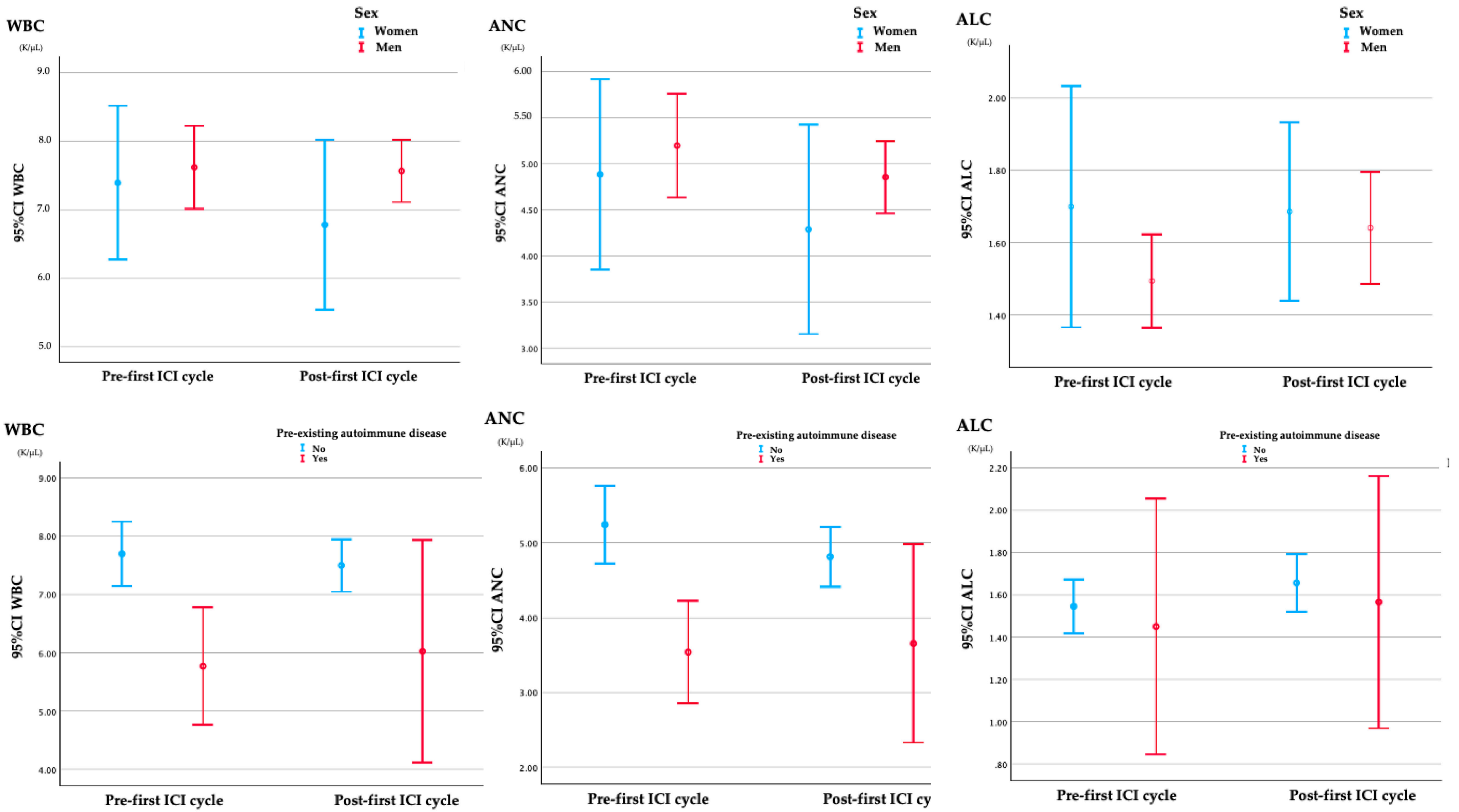

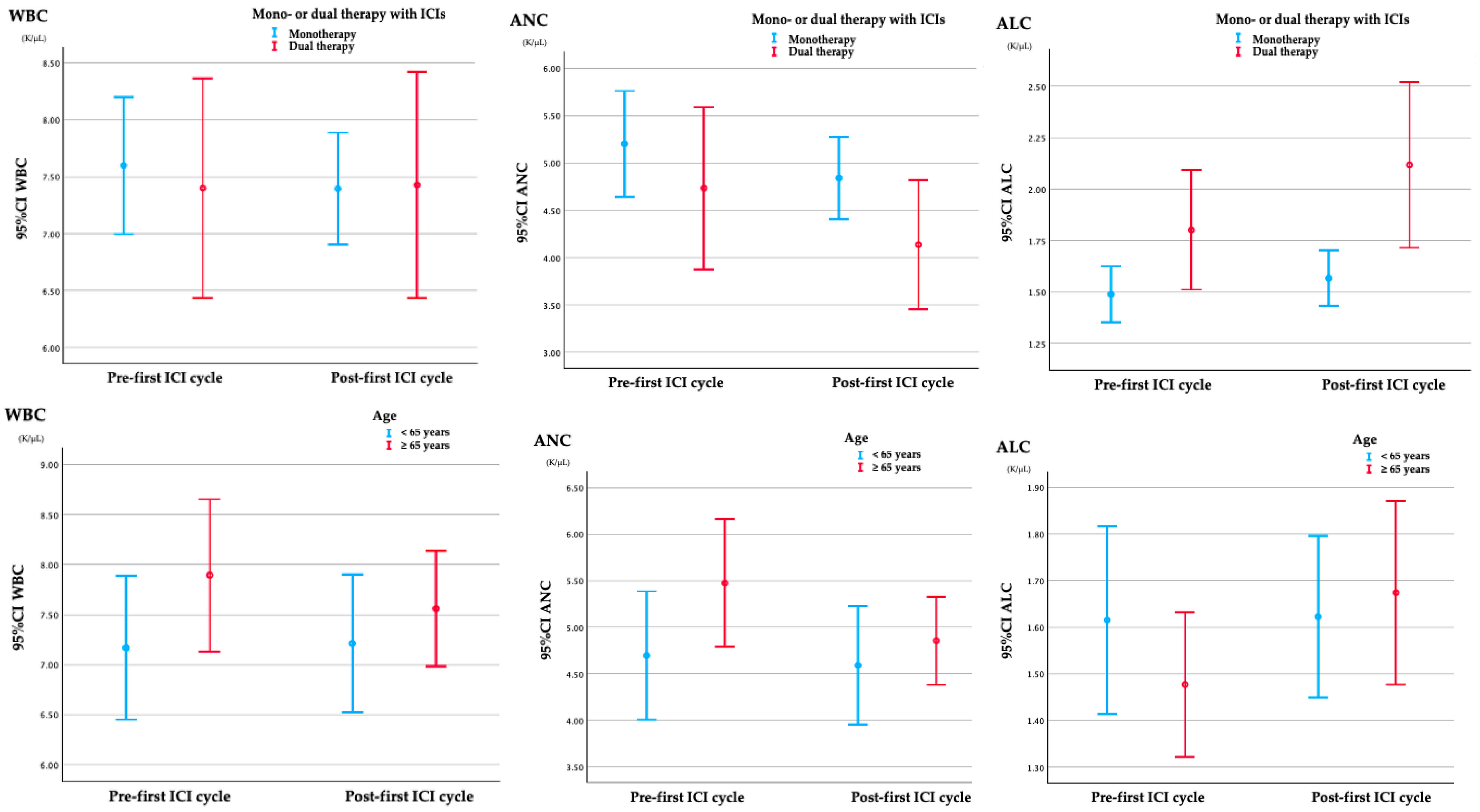

Abbreviations in alphabetical order: ALC, absolute lymphocyte count; ANC, absolute neutrophil count; CI, confidence interval; ICI, immune checkpoint inhibitor; WBC, white blood cell count. Bars represent 95% confidence intervals for the mean.
